# Using Semistructured Telephone Interviews to Collect Qualitative Data From People With HIV Who Are Not in Medical Care: Implementation Study

**DOI:** 10.2196/40041

**Published:** 2022-11-28

**Authors:** Mabel Padilla, Mariana Gutierrez, Jennifer Fagan

**Affiliations:** 1 Division of HIV Prevention Centers for Disease Control and Prevention Atlanta, GA United States; 2 Oak Ridge Institute for Science and Education Division of HIV Prevention Centers for Disease Control and Prevention Atlanta, GA United States

**Keywords:** qualitative research, methods, telephone interviews, HIV, semistructured interviews, recruitment

## Abstract

**Background:**

The Medical Monitoring Qualitative (MMP-Qual) Project was designed to collect qualitative data from people with HIV not engaged in medical care that would complement quantitative data collected by the Medical Monitoring Project (MMP)—a national surveillance system—and inform the MMP’s recruitment and data collection methods.

**Objective:**

Our objectives were to describe the methodology of this project, reflect on the challenges and lessons learned from conducting qualitative telephone interviews at a national level, and describe how we used and plan to use the qualitative data to evaluate our recruitment procedures and quantitative data collection instrument as well as knowledge of HIV care engagement.

**Methods:**

We used stratified purposive sampling to identify and recruit participants who had participated in the structured MMP interview into the MMP-Qual Project. To be eligible, participants must have had an HIV diagnosis, be aged ≥18 years, have lived in an MMP jurisdiction, and have not been engaged in HIV medical care. From August 1, 2018, to May 31, 2019, we conducted semistructured telephone interviews with 36 people with HIV across the United States about several topics (eg, facilitators and barriers to care and experience with surveys). Four trained interviewers conducted semistructured 60-minute telephone interviews with 36 participants. Data collection lasted from August 1, 2018, to May 31, 2019.

**Results:**

From 2018 to 2019, 113 people were eligible to participate in the MMP-Qual Project. Of the people recruited, 28% (22/79) refused to participate. Of those who agreed to participate, 63% (36/57) were interviewed, and 37% (21/57) were no-shows. Of the 34 participants for whom we had complete data, 15 (44%) were aged ≥50 years, 26 (76%) identified as male, 22 (65%) were Black or African American, and 12 (35%) lived in the Southern United States.

**Conclusions:**

We learned that it is possible to obtain rich qualitative data from people with HIV who are not in care via telephone interviews and that this mode might be conducive to talking about sensitive topics. We also learned the importance of flexibility, communication, and coordination because we relied on health department staff to perform recruitment and had difficulty implementing our original sampling strategy. We hope that other projects will learn from our experience conducting qualitative telephone interviews with people with HIV on a national level.

**International Registered Report Identifier (IRRID):**

RR1-10.2196/40041

## Introduction

### Background

Qualitative data can improve and inform quantitative data collection instruments, study recruitment procedures, and our understanding of complex phenomena such as facilitators and barriers to HIV care engagement. According to a systematic review, the practice of using qualitative data for questionnaire development has increased over time, with individual interviews and focus groups being the most common ways to generate questionnaire items [1]. Quantitative data collection instruments that are developed using qualitative methods have survey items that are acceptable, understandable, and relevant and often reflect the perspectives and experiences of the population of interest [2]. Qualitative studies have also been used to inform the recruitment of people with HIV into clinical trials [3,4]. In a qualitative study of facilitators and barriers to recruitment and enrollment of people with HIV with opioid use disorders into a clinical trial, the study staff listed stigma; fear of research; and structural factors such as housing, communication, and transportation as barriers [4]. In another qualitative study, women noted that peer pressure, monetary compensation, and a desire to learn and reflect on their hazardous drinking behavior were reasons for participating in a clinical trial [3]. However, to our knowledge, few qualitative studies have been used to inform the recruitment of people with HIV who are not engaged in care into cross-sectional surveys. Understanding what motivates people with HIV who are out of care to participate in cross-sectional surveys is important because people with HIV who are not retained in care or are unaware of their HIV diagnosis transmit approximately 80% of the annual HIV infections [5]. In addition, qualitative studies have improved our understanding of facilitators and barriers to HIV care engagement. However, most qualitative studies on the subject tend to focus on a single sociodemographic group of people with HIV, are conducted locally, or recruit people from service organizations or infectious disease organizations, thus excluding people with HIV who are not engaged in medical care.

### Objectives

We conducted a qualitative project that would inform and improve the data collection instrument and recruitment procedures of the Medical Monitoring Project (MMP) while also providing rich data on sensitive topics such as HIV care engagement and sexual behaviors. This qualitative project sought to answer the following questions:

1. What facilitators and barriers to HIV care engagement exist among people with HIV who are not in care?
2. How do these facilitators and barriers to HIV care engagement vary by race, region of residence, and length of time for someone who has not been in care?
3. What are the reasons for participating in survey activities for people with HIV who are not in care?

In this paper, we describe our experience implementing this qualitative project in the hopes that others might learn from our experience. In addition, we hope to add to the body of work on experiences using telephone interviews to collect qualitative data. Thus, our objectives were to (1) describe the methodology of this project; (2) reflect on the challenges and lessons learned from conducting qualitative telephone interviews at a national level; and (3) describe how we used, and plan to use, the qualitative data to evaluate our recruitment procedures and quantitative data collection instrument, as well as knowledge of HIV care engagement. Our objectives do not include the discussion of findings from our project because they are reported elsewhere [6]. We felt that doing so would detract from our main objectives.

## Methods

### Overview

The MMP is an annual cross-sectional survey designed to produce nationally representative estimates of the sociodemographic, behavioral, and clinical characteristics of adults with diagnosed HIV in the United States [7]. Sociodemographic and behavioral data are collected through telephone or in-person structured interviews conducted across 23 jurisdictions, and clinical data are collected through medical record abstraction. Since 2015, the MMP has collected quantitative data on people with HIV who are engaged in HIV care as well as those not engaged in HIV care. From 2018 to 2019, we conducted the Medical Monitoring Qualitative (MMP-Qual) Project, which was designed to collect qualitative data from people with HIV not engaged in HIV medical care that would complement quantitative data collected by the MMP and inform the MMP’s recruitment methods and quantitative data collection instrument.

### Sampling

The MMP-Qual Project sample was derived from participants in the MMP’s 2018 data collection cycle: thus, some of the eligibility criteria mirror those of the MMP, including having an HIV diagnosis, being aged ≥18 years, and living in one of the 23 MMP jurisdictions on December 31, 2017. To be eligible to participate in the MMP-Qual Project, participants must have met additional eligibility criteria. Participants must have been out of HIV care for ≥12 months or never received HIV care based on their response to a question on the MMP structured interview. In addition, persons who did not speak English or were incarcerated at the time of the interview were ineligible for participation. We used a stratified purposive sampling strategy to recruit people who had participated in the MMP structured interview into the MMP-Qual Project. We chose this sampling strategy for 2 reasons. First, we wanted to identify differences and similarities in people’s experiences with HIV care. Second, we wanted to ensure that people with certain characteristics were represented in the final sample of the project. A stratified purposive sampling strategy allowed us to do both. In a stratified purposive sampling strategy, the characteristics chosen for stratification are chosen based on the assumption that they offer a unique or important perspective for the phenomenon being investigated [8]. We selected 3 characteristics that we would purposively include in our final sample. These were race or ethnicity, length of time since the last receipt of HIV care, and region of residence at the time of the MMP structured interview. We dichotomized each characteristic into Black participants versus participants who identified as American Indian or Alaska Native, Asian, Native Hawaiian or other Pacific Islander, White, Hispanic or Latino, or multiracial; participants who were out of care for 12 to 23 months versus participants who were out of care for ≥24 months; and participants who lived in the Southern United States versus participants who did not live in the Southern United States. We chose these characteristics because these factors have been related to HIV disparities, and we expected there to be relevant variations in the experiences of people not in HIV care based on these characteristics [5,9-11]. We also reviewed MMP quantitative data (which are nationally representative data of adults diagnosed with HIV in the United States) from prior data collection cycles to determine whether we would have enough participants to interview if we created strata using these 3 characteristics. The data showed that there would be enough participants to interview considering our inclusion and exclusion criteria and these 3 characteristics.

After we decided upon the 3 characteristics that would be included in the final sample, we divided or stratified our sample according to these characteristics. We created a blank nested table with the 3 dichotomized characteristics. This table had 8 cells. The next step was to set a quota for the size of each cell, that is, the number of participants to allocate to each cell or stratum. To do so, we consulted the literature and considered practical realities and concerns. Before we set the quota for each stratum, we discussed what approximate size our final sample should be. According to a study, data saturation had occurred at 12 qualitative interviews [12]. Thus, we wanted a sample size of >12 participants. As we had 8 strata, we wanted to ensure that each stratum had enough participants to generate meaningful data. However, we did not want to select too many participants in each stratum because we had limited resources (eg, time, budget, and staffing). We also used data from past MMP data collection cycles to determine the number of people not engaged in HIV care who were Black versus those who identified as American Indian or Alaska Native, Asian, Native Hawaiian or other Pacific Islander, White, Hispanic or Latino, or multiracial; who lived in the Southern United States versus those who did not live in the Southern United States; and who were out of care for 12 to 23 months versus those who were out of care for ≥24 months. We settled on a quota of 5 participants per stratum after taking all the aforementioned factors into account (Table 1).

As we progressed through the project, we realized that it was becoming increasingly difficult to recruit participants in some of these strata; for example, by December 2018 (ie, 4 months into the project), we had not interviewed any non-Black participant living in the Southern United States who was out of care for 12 to 23 months and any non-Black participant who did not live in the Southern United States who was out of care for ≥24 months. We realized that trying to fill each of these strata—and not interviewing people in strata that had exceeded 5 participants—was impeding our ability to interview enough participants. Thus, halfway through the project (sometime in January 2019) and during data collection, we stopped trying to reach the quota we set for each stratum and simply interviewed whoever was eligible and agreed to participate in the project.

**Table 1:** The stratified purposive sampling strategy for the Medical Monitoring Qualitative Project.

	Length of time without care, months										
	≥12 to ≤23						≥24 or never in care				
Race or ethnicity	Black, non-Hispanic		American Indian or Alaska Native, Asian, Native Hawaiian or other Pacific Islander, White, Hispanic or Latino, or multiracial			Black, non-Hispanic				American Indian or Alaska Native, Asian, Native Hawaiian or other Pacific Islander, White, Hispanic or Latino, or multiracial	
Region	South^a^	Other^b^	South^a^	Other^b^	South^a^			Other^b^	South^a^		Other^b^
Quota (number of participants to interview)	5	5	5	5	5			5	5		5

^a^On the basis of United States Census Bureau classifications: Delaware; Florida; Georgia; Houston, Texas; Mississippi; North Carolina; Texas; and Virginia.

^b^On the basis of United States Census Bureau classifications: California; Chicago, Illinois; Illinois; Indiana; Los Angeles, California; Michigan; New Jersey; New York City, New York; New York; Oregon; Pennsylvania; Philadelphia, Pennsylvania; Puerto Rico; San Francisco, California; and Washington.

### Recruitment

Throughout this paper, the term *interviewers* will refer to staff members in the 23 MMP jurisdictions. They conducted the MMP structured interview with participants and recruited participants into the MMP-Qual Project. The term *CDC* (Centers for Disease Control and Prevention) *interviewers* will refer to CDC staff members who interviewed participants using a semistructured interview guide for the MMP-Qual Project. The CDC interviewers did not have access to participants’ personal information (eg, telephone numbers, addresses, and names); thus, the recruitment strategy for this project relied heavily on interviewers in the 23 jurisdictions who had access to the personal information of the participants who were eligible for the MMP-Qual Project.

At the end of the MMP structured interview, a pop-up message appeared in the computer-assisted personal interview software program notifying the interviewers that a participant was eligible for the MMP-Qual Project based on their responses to a question. After receiving this pop-up message, the interviewers introduced the MMP-Qual Project to eligible participants using a standardized recruitment script. If participants agreed, the interviewers scheduled appointments for the participants to complete a semistructured telephone interview with a CDC interviewer. As CDC interviewers were unable to access participants’ personal information for privacy and confidentiality reasons, interviewers in the jurisdictions were responsible for scheduling interviews, providing appointment reminders, and maintaining the contact information of persons sampled for the MMP-Qual Project. While scheduling interview appointments, the interviewers gave participants a unique code as well as the telephone number to call for the semistructured interview with CDC interviewers. This telephone number was secure and could not be traced to the CDC. Likewise, the CDC interviewers could not see the caller’s telephone number or any other identifying information. The interviewers in the jurisdictions instructed participants to give their unique code to the CDC interviewer upon first contact. This code allowed us to link the data from the semistructured interviews with the MMP quantitative interview and medical record abstraction data.

### Ethical Considerations and Informed Consent

In accordance with the federal human participant protection regulations and guidelines for defining public health research, the MMP was determined to be a nonresearch, public health surveillance activity used for disease control program or policy purposes [13,14]. As this project was determined to not be research, it was not subject to human participant protection regulations, including federal institutional review board review and approval. However, all federal, state, and local MMP staff members adhere to ethical principles and standards by respecting and protecting the privacy, confidentiality, and autonomy of participants. MMP jurisdictions follow their state or local procedures to determine whether the project is subject to state or local human participant protection regulations. Furthermore, MMP data are subject to the CDC’s data security and confidentiality guidelines for HIV, viral hepatitis, sexually transmitted disease, and tuberculosis programs [15]. The security of our data systems meets all Federal Information Systems Management Act, Office of Management and Budget, Department of Health and Human Services, and CDC IT security requirements, which ensure the confidentiality, integrity, and availability of data on federal information systems. Verbal informed consent was obtained from all participants in this project.

### Data Collection

Four trained CDC interviewers conducted semistructured 60-minute telephone interviews with 36 participants. Data collection lasted from August 2018 to May 2019. The interview guide contained 22 questions and prompts that asked about facilitators and barriers to accessing or engaging in HIV medical care, knowledge of HIV treatment as prevention, the preferred method of contact for participation in surveys, and the reason for participation in the MMP. We used information from literature reviews, existing quantitative data from the MMP, and results from the Never in Care Pilot Project to inform the interview guide questions [16]. In addition, community advisory board members for the MMP provided input on interview guide questions and prompts. The community advisory board members are community representatives who are concerned about the well-being of people with HIV in their community and the quality of care that people with HIV are receiving in their jurisdictions. They provide input on the project, including reviewing procedures and methods, ensuring that recruitment methods are effective, providing input on data collection instruments, and ensuring that data collected are helpful to the local community. At the end of every interview, the CDC interviewers asked participants whether they needed additional resources such as referrals to ancillary services or medical care. If participants expressed a need for additional resources, the CDC interviewers informed interviewers in the jurisdictions concerned. The interviewers were then tasked with providing local resources to participants because the CDC interviewers did not have access to local resources or the participants’ contact information.

### Data Analysis

All interviews were audio recorded and transcribed verbatim by 6 trained CDC staff members following a transcription protocol. Data quality checks were performed on all transcripts; for instance, a CDC team member who did not transcribe the transcript under review read the transcript while listening to the audio recording of the interview, ensuring accuracy of transcription and fidelity to the transcription protocol. Five team members independently read 2 interview transcripts and assigned a list of codes that were used to develop the initial codebook. The team applied the initial codebook to 2 more interview transcripts and continued until they reached consensus. The team performed intercoder reliability on 21% (7/34) of the transcripts, reviewing and discussing codes with κ coefficients <0.61 until reaching agreement on code application. The team also established trustworthiness (eg, credibility and dependability) and rigor through various means; for instance, we kept records of interview notes; created thorough documentation of all project processes, including notes of code and theme generation and chronology of activities; maintained documentation of team meetings; and stored data and notes in well-organized archives. In addition, we engaged in reflexivity—documenting and discussing how our understanding might have affected the data analysis process—and peer debriefing after the interviews [17,18]. We used applied thematic analysis as the primary qualitative data analysis method [19].

## Results

### Interview Data

**Figure 1:**
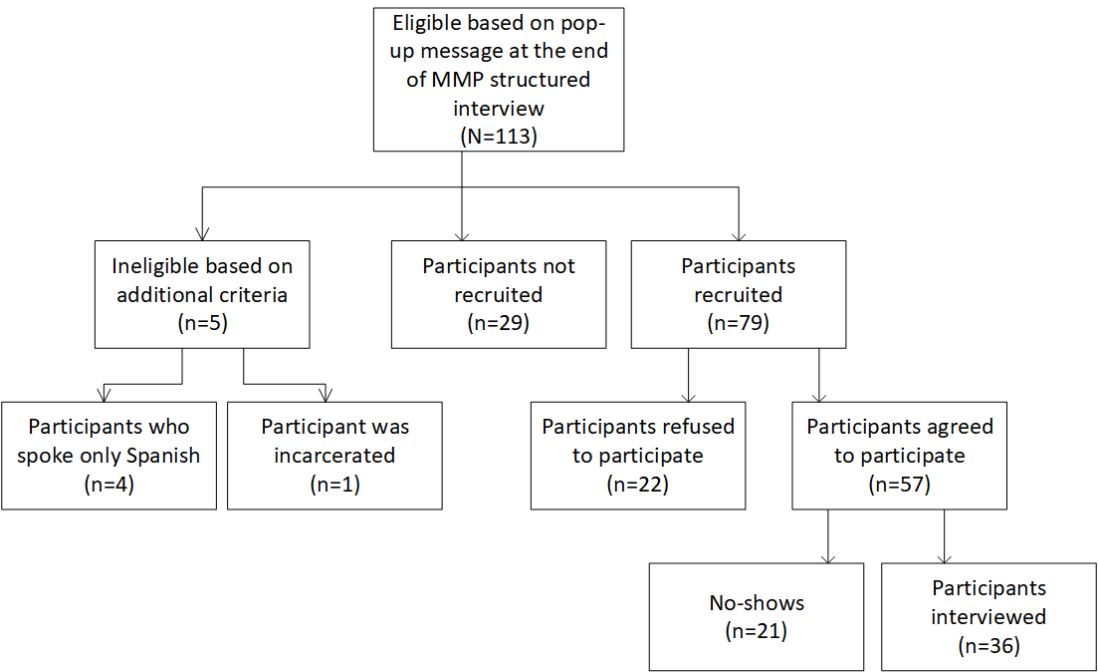
Flow diagram of participant enrollment. MMP: Medical Monitoring Project.

From 2018 to 2019, a total of 113 MMP participants were eligible for participation in the MMP-Qual Project based on their responses to a question on the MMP structured interview. Of the 113 people, 5 (4.4%) were ineligible because they were incarcerated (n=1, 20%) or spoke only Spanish (n=4, 80%). Of the 113 people, 29 (25.7%) were not recruited: in most of these cases, the interviewers missed the pop-up message at the end of the structured interview and thus failed to recruit the participant, whereas some people were not recruited for reasons not given. Of the 113 people, 79 (69.9%) were recruited, of whom 22 (28%) refused to participate, and 57 (72%) agreed to participate. However, of the 57 people who agreed to participate, 21 (37%) were no-shows, that is, they never called the CDC to do the interview and would be considered soft refusals. Thus, of the 57 people who agreed to participate, 36 (63%) were interviewed. Of these 36 interviewees, we had complete data for 34 (94%); of the 36 interviewees, 1 (3%) stated that they were HIV negative, which meant we had to end the interview, and the interview with 1 (3%) participant was never audio recorded (Figure 1).

Of the 23 jurisdictions participating in the MMP-Qual Project, 16 (70%) completed interviews, whereas 7 (30%) did not complete any interviews. The jurisdictions that did not complete interviews were Illinois; Los Angeles, California; Mississippi; New York; Pennsylvania; Puerto Rico; and Texas. Among these jurisdictions, Illinois and Mississippi only had refusals; Los Angeles, California, and Pennsylvania did not recruit participants; Puerto Rico had participants who were ineligible because of language; and New York and Texas had participants who were no-shows.

### Demographic Data

Of the 34 participants with complete interviews, 15 (44%) were aged ≥50 years, 10 (29%) were aged 18 to 39 years, and 9 (26%) were aged 40 to 49 years. Of the 34 participants, 26 (76%) identified as male (both sex assigned at birth and gender identity were male), and 7 (21%) identified as female (both sex assigned at birth and gender identity were female). We had missing gender identity data for 3% (1/34) of the participants (we had data related to the sex assigned at birth but no gender identity data). Of the 34 participants, 22 (65%) were Black or African American, and 7 (21%) were non-Hispanic White. We classified 8% (3/34) of the participants as another race or ethnicity, which included American Indian or Alaska Native, Native Hawaiian or other Pacific Islander, or multiracial, and 6% (2/34) identified as Hispanic or Latino. Of the 34 participants, 12 (35%) lived in the South, 8 (24%) lived in the Midwest, 7 (21%) lived in the West, and 7 (21%) lived in the Northeast (Table 2).

Of the 34 participants, 12 (35%) requested linkage to care or referrals to ancillary services at the end of the qualitative interview; 4 (12%) requested financial assistance such as social security disability insurance, supplemental security income, and financial assistance for copays and medications; 2 (6%) requested housing and food or meal services; 4 (12%) requested referrals to an HIV medical provider; and 5 (15%) requested other medical care, including referrals to a dentist, optometrist, and psychiatrist.

**Table 2:** Demographic characteristics of participants in the Medical Monitoring Qualitative Project (N=34).

Characteristics^a^			Values, n (%)
**Age (years)**			
	18 to 39	10 (29)	
	40 to 49	9 (26)	
	≥50	15 (44)	
**Gender^b^**			
	Male	26 (76)	
	Female	7 (21)	
	Transgender	0 (0)	
	Missing^c^	1 (3)	
**Race and ethnicity**			
	Non-Hispanic Black or African American	22 (65)	
	Hispanic or Latino^d^	2 (6)	
	Non-Hispanic White	7 (21)	
	Another race or ethnicity^e^	3 (8)	
**Current US region of residence^f^**			
	West	7 (21)	
	Midwest	8 (24)	
	Northeast	7 (21)	
	South	12 (35)	

^a^Participant demographic data were obtained from the Medical Monitoring Project structured interview.

^b^Gender was based on gender identity and sex assigned at birth. Transgender persons were defined as those who self-identified as transgender or who reported a gender identity different from sex assigned at birth.

^c^Data were coded as missing because participants refused to answer.

^d^Hispanic or Latino persons may be of any race. Persons are classified into only 1 race or ethnicity category.

^e^Includes American Indian or Alaska Native, Asian, Native Hawaiian or other Pacific Islander, or multiracial.

^f^Regions based on classification by United States Census Bureau and limited to Medical Monitoring Project jurisdictions: West (California, Oregon, and Washington), Midwest (Indiana, Illinois, and Michigan), Northeast (New Jersey, New York, and Pennsylvania), and South (Delaware, Florida, Georgia, North Carolina, Virginia, and Texas).

## Discussion

### Overview

This paper describes the methodology of the MMP-Qual Project in the hopes that others might learn from our experience. We experienced several challenges along the way: our initial sampling strategy was difficult to implement, given our practical realities; our recruitment strategy relied on our health department colleagues (who were geographically dispersed); and our mode of data collection (ie, telephone interviews), although practical, might have created barriers to forming trust and building rapport with the participants. Despite these challenges, we were able to collect data from a diverse group of people with HIV. We have used some of these data to report on facilitators and barriers to HIV care engagement, improve our MMP data collection instrument, and inform MMP recruitment procedures [6]. In the following sections, we describe in more detail the challenges we faced and the lessons we learned.

### Challenges During Recruitment and Data Collection

Although our goal was to interview 20 Black and 20 non-Black participants, we were only able to interview 12 non-Black participants. Although our goal was to interview 20 people who lived in the Southern United States and 20 people who did not live in the Southern United States, we were only able to interview 12 people who lived in the Southern United States. Finally, although our goal was to interview 20 people who were out of care for 12 to 23 months and 20 people who were out of care for ≥24 months, we only interviewed 14 people who were out of care for ≥24 months. Upon reflection, we believe that there were several reasons why we were not able to meet our goals. First, although we chose our sampling strategy partly because of practical reasons (such as how much time and money we had), it was only during recruitment and data collection that other practical realities came to light, including a delayed project start because of technical issues with scheduling. Furthermore, we realized that we selected many stratification criteria. The more stratification criteria one includes in one’s sample frame, the more difficult recruitment becomes and the longer it takes to find participants [20]. Thus, we might have found more success if we had selected fewer (ie, 1 or 2) stratification criteria. We also believe that we might have overestimated people’s interest in participating in the MMP-Qual Project. We assumed that if participants had taken part in the MMP structured interview, they probably would participate in an additional qualitative component of the project. However, the 2 activities (the MMP structured interview and the MMP-Qual Project) were different, including data collection being conducted by 2 different institutions (the local jurisdiction vs the CDC). Participants might have felt skeptical about being interviewed by CDC interviewers and thus declined to participate in the qualitative project; for instance, during a qualitative interview, a participant said they were hesitant and concerned about being interviewed by a CDC staff member. Despite these challenges, we felt that we obtained data from a diverse array of participants with regard to age, gender, race and ethnicity, and region of residence.

Another challenge we faced was being unable to recruit people into the MMP-Qual Project ourselves, leading to our having to rely on staff in the 23 jurisdictions to recruit participants and schedule interviews. Interviews had to be scheduled in advance, and participants could not complete the qualitative interviews on the same day as, or right after, the MMP structured interview; thus, participants had to be flexible to meet our schedules as much as we had to be flexible to meet theirs. Sometimes CDC interviewers and health department interviewers were in different time zones. If a participant lived on the west coast and wanted to schedule an interview late in the day (eg, after 9 PM), that would not be possible for CDC interviewers who were on the east coast and did not have access to their offices during certain hours (eg, after midnight). Thus, interview slots were sometimes limited because of time zone differences and limited resources (eg, available CDC interviewers). In addition, before CDC interviewers could interview a participant, they needed the participant’s unique code, which interviewers in the jurisdictions had assigned to participants during recruitment. Because of strict data security and confidentiality procedures, interviewers in the jurisdictions needed to send this code to the CDC through a secure system, a process that sometimes took several hours. Another option that interviewers in the jurisdictions had was to call the CDC to verbally provide the unique code. If we did not receive the code, we could not interview the participant because we had no way of confirming their identity. This was challenging because it required consistent communication and coordination with interviewers in the jurisdictions who had other competing priorities. To address these challenges, we conducted training with all interviewers across the 23 jurisdictions on recruitment procedures for the MMP-Qual Project, which included being aware of the pop-up message indicating eligibility, assigning eligible participants a unique code that would be linked to the quantitative data, scheduling interviews, and the importance of sending the unique code promptly to the CDC. If we were to conduct a similar project again, we would dedicate more time to developing a more efficient electronic scheduling system that would allow interviewers in the jurisdictions to share the participant’s unique code during scheduling.

CDC interviewers did not have access to participants’ personal information such as names and telephone numbers. Although this was key to ensuring and maintaining anonymity during the interview process, it also brought on several challenges. For one, if a person failed to call the CDC for their scheduled interview, CDC interviewers could not contact them to reschedule. This required continual communication with interviewers in local jurisdictions who performed the recruitment because they were responsible for rescheduling interviews and maintaining contact with eligible participants. In addition, it was challenging to establish rapport with the participant if we could not address them by name. We stressed that they should not reveal their name, location, health care provider’s name, or any personally identifiable information. Despite these challenges, we believe that the anonymous nature of the interview allowed participants to disclose sensitive information about topics that many of them discussed feeling stigmatized by. Furthermore, because the telephone we used had minimal technology (this was done to safeguard the confidentiality of participants), we could not directly record the interviews using the telephone. Thus, we used an audio recorder to record the interview, which required the use of the speaker option on the telephone. This made the audio quality of interview recordings suboptimal at times. When audio quality was poor, and we could not discern what the participant was saying, we transcribed segments of text from the interview as inaudible, which meant the loss of these data.

Finally, we realize that the interview guide we used was lengthy. The interview guide covered 4 main topics and included an opening and closing question. It covered 22 questions, not including probes, in 60 minutes. If we were to conduct a project like this again, we might ask fewer semistructured interview questions because participants might have felt fatigued or had insufficient time to provide robust responses to some questions (especially those posed toward the end). We also could have modified the interview guide halfway through the project to reduce the number of questions and thereby participant burden. However, we felt that the interview length issue was mitigated because the questions on each topic were similar—and sometimes participants naturally answered questions that were about to be asked. Furthermore, CDC interviewers received training on what to do if an interview was not moving at the pace required to ask all 22 questions and how to determine whether a participant had adequately addressed questions that had not been posed yet; for instance, sometimes, interviewers skipped questions that had already been addressed in some form during prior questions. For the most part, CDC interviewers did not feel rushed during the interviews and obtained robust data from the participants.

### Reflections

We described the methodology of the MMP-Qual Project and described the recruitment and data collection challenges. We learned several lessons from our experience. One of the first challenges we faced was having to pivot from our original sampling strategy. We needed to be flexible based on how recruitment was progressing in the field and cognizant of timelines and resource constraints. Although we did not meet our initial sampling goals, we obtained perspectives from a diverse group of people with HIV who nonetheless shared common experiences relating to not being engaged in HIV care.

In addition, recruitment and interviews were being conducted by different staff in different states: interviewers in local jurisdictions recruited participants, whereas CDC interviewers conducted the semistructured interviews. To ensure successful recruitment, communication, coordination, and training were key. We trained interviewers on how to recruit and checked with staff in the jurisdictions about recruitment progress regularly. Interviewers who recruited eligible participants for the MMP-Qual Project were typically the same interviewers who had conducted the structured MMP interview with the participant. Thus, interviewers in the jurisdictions had already established rapport with participants during the structured interview. At the end of the qualitative interviews, participants expressed having been more open to participating in the MMP-Qual Project based on their experience with the structured interview. Other qualitative projects attempting to sample participants from a cross-sectional survey might also experience this benefit.

This was also the first time we conducted telephone qualitative interviews for the MMP. As a mode of qualitative in-depth data collection, the telephone has become a practical option for qualitative research: telephone interviews allow for data collection among geographically dispersed participants, reduce cost compared with in-person interviews, and increase privacy for participants [21,22]. In addition, telephone interviews give participants greater anonymity than in-person interviews, which increases feelings of privacy, and this may be particularly important when sensitive questions are asked and the need for anonymity is high, which is often the case for people with HIV [21,23]. By contrast, researchers posit that it is difficult to form trust and build rapport during qualitative telephone interviews because of the physical separation between participant and interviewer, which may compromise the richness and quality of the data. However, there is minimal evidence to indicate that data quality is compromised when using telephone interviews [24]. In a study using qualitative telephone interviews to understand hazardous drinking among sexual minority women who had participated in the population-based National Alcohol Survey, participants provided rich narrative data over the telephone about sensitive topics such as sexual identity, traumatic experiences, and alcohol or drug use [22]. We similarly collected rich data from our participants. We believe that the anonymous nature of the interviews allowed participants to disclose sensitive information about a topic that many participants discussed feeling stigmatized by (Textbox 1). The telephone as a mode of qualitative data collection was also an affordable option for our project and reduced participant burden because participants did not have to travel to a specific location to be interviewed.

We were able to obtain rich data by completing training with CDC interviewers who conducted the qualitative interviews, which included mock interviews, and by using strategies to build rapport, such as those detailed by Drabble et al [22]. Throughout the qualitative interviews, interviewers used orienting statements to let participants know what to expect during the process and emphasized the voluntary nature of their participation (eg, they did not have to answer any question they did not want to answer). Interviewers could only respond to auditory cues (such as tone of voice or background noise) and the content of the answers; thus, their active listening skills were heightened. In addition, interviewers used neutral words such as “sure,” “okay,” “I see,” or “yeah” to vocalize that they understood and were listening. Finally, interviewers communicated their appreciation to the participants and maintained an accepting and nonjudgmental tone, which is key when discussing HIV.

In the MMP quantitative data collection instrument, we ask participants about facilitators and barriers to HIV care. We used the data from the qualitative interviews to evaluate whether our current questions on facilitators and barriers to care adequately capture the most salient barriers in the lives of participants who are not in care. On the basis of the qualitative data, we confirmed that the most salient barriers, including stigma, patient-provider relationships, and mental health issues, were captured in our data collection instrument. Thus, the MMP data collection instrument reflects the perspectives and experiences of people with HIV who are not engaged in care. Other cross-sectional surveys might be interested in conducting qualitative projects to understand the extent to which their data collection instruments reflect the experiences of their populations of interest. In the qualitative interviews, we also asked participants about their preferred method (eg, telephone call, letter, email, or in person) of contact for participation in surveys, concerns about participation in the MMP, the reason for participation in the MMP, and whether they would complete the MMP quantitative interview on the web. We are using these data to inform recruitment procedures and materials (eg, recruitment scripts, recruitment letters, and website materials).

**Challenges and lessons learned from conducting the Medical Monitoring Qualitative Project.**

- Recruitment of participants to fill each quota in our stratified purposive sampling strategy
  Pivoting from your original sampling strategy based on how recruitment progresses in the field as well as on timelines and resource constraints
  Reviewing incoming data throughout data collection to ensure that you are obtaining data from a diverse array of participants as it relates to characteristics important to your evaluation and research questions
  Selecting fewer stratification criteria if there are limited resources

- Being unable to recruit participants ourselves and relying on health department staff across the country
  Maintaining consistent communication and coordination with staff who perform recruitment
  Training staff who perform recruitment on eligibility criteria, security, confidentiality, and scheduling interviews
  Creating an efficient electronic scheduling system when scheduling interviews across different geographic locations
  Tapping into any existing relationship with staff: participants may be more open to participating in a qualitative project after having built rapport with local staff during recruitment and previous participation in a survey

- No access to participants’ personal information (eg, names and telephone numbers)
  Recognizing that the anonymous nature of interviews may allow participants to disclose sensitive information, as they might feel stigmatized by the condition

- Being unable to directly record interviews using the telephone (requiring the use of an audio recorder and speakerphone, which made audio quality suboptimal at times)
  Investigating telephone technology that would allow you to record telephone interviews without obtaining any personal information (eg, names and phone numbers)

- The first time we used telephone interviews to collect qualitative data for the Medical Monitoring Project
  Training interviewers who conduct qualitative interviews to navigate the different sets of challenges and nuances posed by telephone interviews
  Using strategies to build rapport during telephone interviews (including orienting participants to the interview process, emphasizing the voluntary nature of participation, using neutral words to vocalize understanding, and maintaining an accepting and nonjudgmental tone)

### Conclusions

The MMP-Qual Project was conducted to complement quantitative data collected in the MMP and to inform and improve the MMP’s recruitment procedures and data collection instrument. We experienced several challenges during our project, but we also learned many lessons. We learned to be flexible based on recruitment progress in the field, to use live web-based training to train interviewers on participant recruitment into a qualitative project, to build rapport during qualitative telephone interviews, and to safeguard the privacy and confidentiality of our participants. We learned that it is possible to obtain rich qualitative data over the telephone from people with HIV who are not engaged in HIV medical care and that this mode might be particularly helpful for such a sensitive topic.

## Abbreviations

CDC: Centers for Disease Control and Prevention
MMP: Medical Monitoring Project
MMP-Qual: Medical Monitoring Qualitative

## Data Availability

National Medical Monitoring Project (MMP) data are not publicly available because of the need for specialized technical assistance for working with the large and complex data sets and the security and confidentiality guidelines for the release of HIV surveillance data. However, the Centers for Disease Control and Prevention (CDC) will grant access to MMP data in accordance with security and confidentiality guidelines on a case-by-case basis. Researchers may submit analysis concept proposals that are reviewed and prioritized based on their importance for public health, their scientific merit, and on the needs and current workload of the team that oversees the MMP at the CDC. There are currently no fees associated with accessing or receiving MMP data, but the release of data is subject to the availability of CDC resources to complete such requests. More information on the appropriate procedures for concept proposals can be obtained by contacting the CDC [25].

## References

[1] Ricci L, Lanfranchi J, Lemetayer F, Rotonda C, Guillemin F, Coste J, Spitz E (2019). Qualitative methods used to generate questionnaire items: a systematic review. Qual Health Res.

[2] McKenna S, Meads D, Doward L, Twiss J, Pokrzywinski R, Revicki D, Hunter CJ, Glendenning GA (2011). Development and validation of the living with chronic obstructive pulmonary disease questionnaire. Qual Life Res.

[3] Canidate S, Cook C, Varma D, Carnaby G, Ennis N, Stetten N, Cook RL (2020). Recruitment, experience, and retention among women with HIV and hazardous drinking participating in a clinical trial. BMC Public Health.

[4] Hoffman K, Baker R, Kunkel L, Waddell E, Lum P, McCarty D, Korthuis PT (2019). Barriers and facilitators to recruitment and enrollment of HIV-infected individuals with opioid use disorder in a clinical trial. BMC Health Serv Res.

[5] Li Z, Purcell DW, Sansom SL, Hayes D, Hall HI (2019). Vital signs: HIV transmission along the continuum of care - United States, 2016. MMWR Morb Mortal Wkly Rep.

[6] Padilla M, Carter B, Gutierrez M, Fagan J (2022). The boundary of HIV care: barriers and facilitators to care engagement among people with HIV in the United States. AIDS Patient Care STDS.

[7] Beer L, Johnson CH, Fagan JL, Frazier EL, Nyaku M, Craw JA, Sanders CC, Luna-Gierke RE, Shouse RL (2019). A national behavioral and clinical surveillance system of adults with diagnosed HIV (the medical monitoring project): protocol for an annual cross-sectional interview and medical record abstraction survey. JMIR Res Protoc.

[8] Palinkas LA, Horwitz SM, Green CA, Wisdom JP, Duan N, Hoagwood K (2015). Purposeful sampling for qualitative data collection and analysis in mixed method implementation research. Adm Policy Ment Health.

[9] (2022). HIV in the United States by region. Centers for Disease Control and Prevention.

[10] (2021). HIV Surveillance Report. Centers for Disease Control and Prevention.

[11] Dasgupta S, Oster A, Li J, Hall H (2016). Disparities in consistent retention in HIV care--11 states and the district of Columbia, 2011-2013. MMWR Morb Mortal Wkly Rep.

[12] Guest G, Bunce A, Johnson L (2016). How many interviews are enough?. Field Method.

[13] (2021). 45 CFR 46. Office for Human Research Protection.

[14] Distinguishing public health research and public health nonresearch. Centers for Disease Control and Prevention.

[15] (2011). Data security and confidentiality guidelines for HIV, viral hepatitis, sexually transmitted disease, and tuberculosis programs: standards to facilitate sharing and use of surveillance data for public health action. National Center for HIV/AIDS, Viral Hepatitis, STD, and TB Prevention.

[16] Bertolli J, Garland PM, Valverde EE, Beer L, Fagan JL, Hart C, Never in Care Pilot Project Team (2013). Missed connections: HIV-infected people never in care. Public Health Rep.

[17] Morse J, Barrett M, Mayan M, Olson K, Spiers J (2016). Verification strategies for establishing reliability and validity in qualitative research. Int J Qual Method.

[18] Lincoln Y, Guba E (1986). But is it rigorous? Trustworthiness and authenticity in naturalistic evaluation. New Direction Program Eval.

[19] Guest G, MacQueen K, Namey E (2012). Applied Thematic Analysis.

[20] Trost JE (1986). Statistically nonrepresentative stratified sampling: a sampling technique for qualitative studies. Qual Sociol.

[21] Block E, Erskine L (2012). Interviewing by telephone: specific considerations, opportunities, and challenges. Int J Qual Method.

[22] Drabble L, Trocki K, Salcedo B, Walker P, Korcha R (2016). Conducting qualitative interviews by telephone: lessons learned from a study of alcohol use among sexual minority and heterosexual women. Qual Soc Work.

[23] Mealer M, Jones Rn J (2014). Methodological and ethical issues related to qualitative telephone interviews on sensitive topics. Nurse Res.

[24] Novick G (2008). Is there a bias against telephone interviews in qualitative research?. Res Nurs Health.

[25] Medical Monitoring Project (MMP). Centers for Disease Control and Prevention.

